# Introducing Mediterranean Lupins in Lamb Diets: Effects on Carcass Composition, Meat Quality, and Intramuscular Fatty Acid Profile

**DOI:** 10.3390/ani12141758

**Published:** 2022-07-08

**Authors:** Mariana Almeida, Sofia Garcia-Santos, Daniela Carloto, André Arantes, Jose M. Lorenzo, José António Silva, Virgínia Santos, Jorge Azevedo, Cristina Guedes, Luís Ferreira, Severiano Silva

**Affiliations:** 1 Veterinary and Animal Research Centre (CECAV), University of Trás-os-Montes e Alto Douro, Quinta de Prados, 5000-801 Vila Real, Portugal; jasilva@utad.pt (J.A.S.); vsantos@utad.pt (V.S.); jazevedo@utad.pt (J.A.); cguedes@utad.pt (C.G.); ssilva@utad.pt (S.S.); 2 Associate Laboratory for Animal and Veterinary Sciences (AL4AnimalS), University of Trás-os-Montes e Alto Douro, Quinta de Prados, 5000-801 Vila Real, Portugal; 3 Department of Animal Science, University of Trás-os-Montes e Alto Douro, Quinta de Prados, 5000-801 Vila Real, Portugal; daniela30121996@hotmail.com (D.C.); arantex@hotmail.com (A.A.); lmf@utad.pt (L.F.); 4 Centre for the Research and Technology Agro-Environmental and Biological Sciences (CITAB), University of Trás-os-Montes e Alto Douro, Quinta de Prados, 5000-801 Vila Real, Portugal; ssantos@utad.pt; 5 Centro Tecnológico de la Carne de Galicia, Rúa Galicia N°4, Parque Tecnológico de Galicia, San Cibrán das Viñas, 32900 Ourense, Spain; jmlorenzo@ceteca.net; 6 Área de Tecnología de los Alimentos, Facultad de Ciencias de Ourense, Universidad de Vigo, 32004 Ourense, Spain

**Keywords:** lupins, lambs, meat quality, pH, color, carcass characteristics, fatty acids

## Abstract

**Simple Summary:**

The main aim of this preliminary study was to evaluate the effects of replacing soybean meal with lupins on carcass traits, meat characteristics, meat characteristics, and meat fatty acid profile in lambs. Two trials were conducted: In trial 1, the soybean meal was partially replaced by *Lupinus albus* or *Lupinus luteus*; in trial 2, lambs were fed four diets with graded levels of *Lupinus luteus*, ranging from 0 to 200 g/kg. The lambs were slaughtered to evaluate carcass characteristics, meat composition, and fatty acids profile. Carcass composition was not affected (*p* > 0.05) by diet in both trials. Meat quality attributes did not vary (*p* < 0.05) between trials 1 and 2. Overall, fatty acid content was not affected by diet (*p* > 0.05) in both trials. Soybean meal produced the same results as lupins in this study, indicating the latter as a potential alternative protein source, although research should focus on meat palatability.

**Abstract:**

The objective of this preliminary study was to evaluate the effects of partial replacement of soybean meal by lupins on lambs’ diets, on the carcass traits, meat characteristics, and meat fatty acid profile. Two trials were conducted: In trial 1, the soybean meal (control; C) was partially replaced by *Lupinus albus* or *Lupinus luteus* (50 g/kg; LA5 and LL5, respectively); in trial 2, lambs were fed four diets with graded levels of *Lupinus luteus* (0, 100, 150 and 200 g/kg; C, LL10, LL15, LL20, respectively). At the end of the feeding trials, animals were slaughtered to evaluate carcass characteristics and meat composition, including fatty acids. Carcass composition in tissues was not affected (*p* > 0.05) by diet in both trials. Additionally, no significant (*p* < 0.05) differences were observed in meat quality attributes between diets in trials 1 and 2. Overall, the *Longissimus* muscle’s fatty acid content was not affected by diet (*p* > 0.05) in both trials. Carcass and meat quality was overall comparable between lambs fed with soybean meal and lupins, indicating the latter as a potential alternative protein source. However, the lack of significant differences could also be attributed to the small sample size.

## 1. Introduction

Over the last decade, the European Union (EU) has focused on solving its dependency on imported soybean for animal feeding [1]. Different protein alternatives have been evaluated, such as faba beans (*Vicia faba*), peas (*Pisum sativum*), and lupins (*Lupinus* spp.). These species are well-adapted to the Mediterranean climate and soil characteristics [1,2]. Although these Mediterranean legumes species offer lower protein contents than soybean meal, which is the most common protein source [3], they represent a local solution and possible replacement candidate for soybean meal in livestock feeding [4,5]. The inclusion of lupins as an alternative protein source in ruminant feeding has shown positive results in ewe milk production and composition, and it improved nursing performance [6,7], resulting in similar values to those obtained with soybean meal diets. Most important are the results found on carcass traits and meat quality of lambs fed with lupins [8,9,10], which are very encouraging and again indicate that this might be an adequate substitute. Facciolongo et al. [8] found comparable results on carcass traits between Awassi lambs fed soybean meal and lupins, which is in accordance with more recent results found in Gentile di Puglia lambs [9]. Overall, the inclusion of legume grains in ruminant feeding, lupins in specific, usually provides very similar results in meat quality and carcass traits to the ones obtained with soybean meal, as previously stated. Most studies, however, present results on the influence of *Lupinus albus* (white lupin) incorporation on ruminant diets, and few studies on *Lupinus luteus* (yellow lupins) incorporation in finishing lambs’ diets have been published, although this inclusion has provided comparable results to those from a soybean-meal-based diet [11]. Although there has been a decline in lamb meat production and consumption in Portugal during the last decade [12], sheep are extremely well-adapted to the Mediterranean diverse production systems and could benefit from replacing soybean meal with lupins [13], especially yellow lupins which used to be cultivated in the area. This study provides results on the inclusion of yellow lupins in lambs’ diets, which is not common and could provide a relevant step toward broader studies with legume grains. After analyzing the inclusion of *Lupinus albus* and *Lupinus luteus* in *Churra da Terra Quente* lambs’ diets and its effects on performance [14], this study aimed to evaluate the effects of this inclusion on carcass composition and meat quality. 

## 2. Materials and Methods

Two different trials were conducted over two consecutive years to study the effect of introducing Mediterranean lupins in diets on lamb’s carcass composition and meat quality. The first trial evaluated the introduction of low percentages of *Lupinus albus* and *Lupinus luteus*, while the second one focused on increasing levels of *Lupinus luteus*. Both trials were held at the University of Trás-os-Montes and Alto Douro (UTAD) at Vila Real (Portugal). Daily handling was performed by trained personnel while respecting the Portuguese law on animal welfare in experimental research [15]. The protocol was approved by the ORBEA (Animal Welfare Body) of the University (669-e-DZ-2018).

### 2.1. Animals and Housing

A total of 28 Churra da Terra Quente weaned male lambs were used in the trials. In the first trial, 12 lambs with ages between 92 and 110 days and initial body weight (BW) of 18 ± 2.8 kg were distributed between 3 groups of 4 animals each. In trial 2, 16 more animals were added, so a total of 16 lambs (16 ± 2.6 kg BW and 92–110 days of age) were split into 4 groups of 4 animals each. Both trials started 3 weeks after weaning a 21-day adaptation period and lasted 12–16 weeks.

### 2.2. Diets

**Figure 1:**
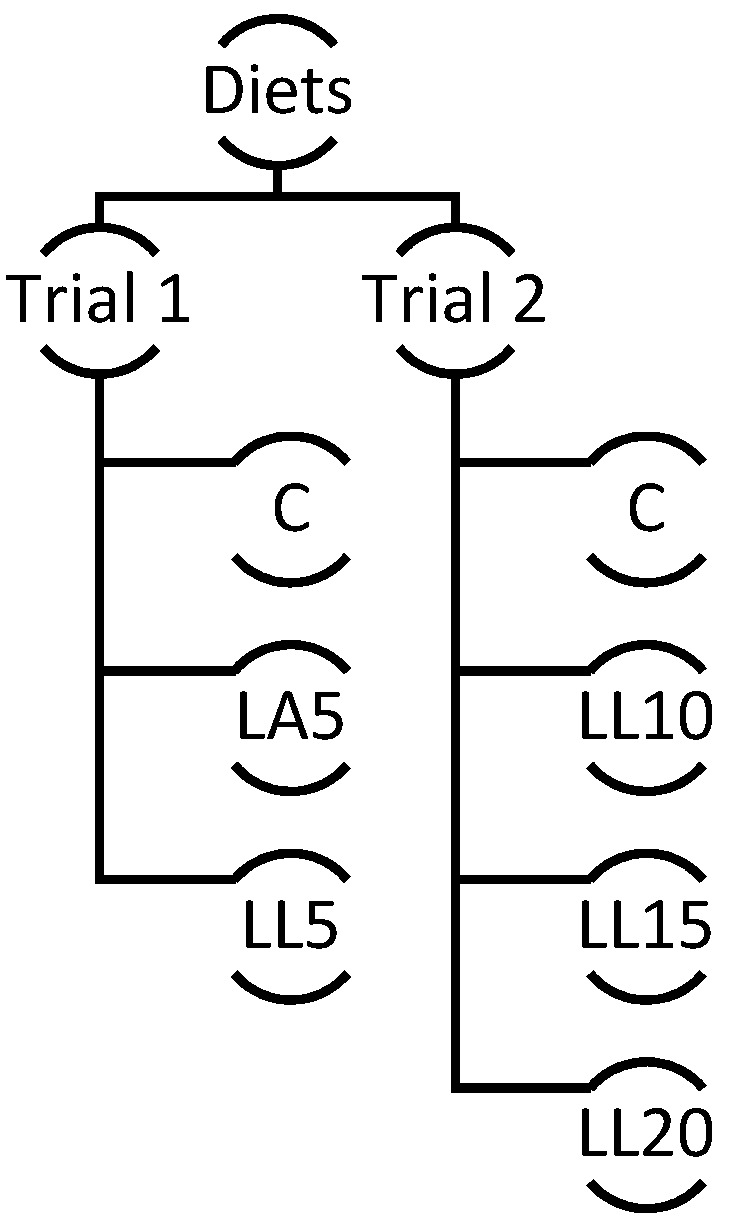
Diagram showing the distribution of diets by trial 1 and trial 2 with an indication of the level of the incorporation of *Lupinus albus* (LA) and Lupinus luteus (LL). C—control; LL5—*Lupinus luteus* 50 g/kg; LA5—*Lupinus albus* 50 g/kg; LL10—*Lupinus luteus* 100 g/kg; LL15—*Lupinus luteus* 150 g/kg; LL20—*Lupinus luteus* 200 g/kg; DM.

During the trials, each group received a different diet (Figure 1). Three diets were provided in trial 1: a control diet (C; 150 g/kg soybean meal) without lupin incorporation, a diet with 50 g/kg *Lupinus luteus* cv. Mister (LL5), and a diet with 50 g/kg *Lupinus albus* cv. Nacional (LA5). In trial 2, the lambs were fed four different diets: one group was provided with the control diet (C; 170 g/kg soybean meal), and the others consumed diets with different incorporations of *Lupinus luteus* cv. Mister (100 g/kg, 150 g/kg, 200 g/kg; LL10, LL15, LL20). 

Details on diet formulation and chemical composition are found in Table 1, which is published by Almeida et al. [14].

### 2.3. Carcass Traits, Cutting, and Dissection

Lambs were weighed weekly until they reached their target weight and then were submitted to a digestibility trial before slaughter, as described in Almeida et al. [14]. At the end of this trial, the animals were slaughtered at 23 ± 2 kg live weight using standard commercial procedures according to the Portuguese law on animal welfare in experimental research [15]. In trial 1, three animals per group were slaughtered. In trial 2, all animals were slaughtered except one from the control group that died at the end of the digestibility trial. Live weight at slaughter (LWS) was recorded after an overnight fast. Carcass dressing and dissection were performed after the methods of Fisher and DeBoer [16]. Carcasses were refrigerated for 24 h at 4 °C, and cold carcass weight (CCW) was recorded. The carcass dressing percentage was calculated on a CCW basis. The carcasses were then split along the vertebral column and the left side was divided into eight commercial cuts following the procedure of Santos et al. [17]. After weighing, each cut was dissected into muscle, bone, fat (subcutaneous and intermuscular fat), and residues (major blood vessels, ligaments, tendons, and thick connective tissue sheets associated with some muscles). Experienced operators performed the dissection in a controlled environment with room temperature below 20 °C.

### 2.4. Muscle Sampling

The Longissimus thoracis et lumborum muscle (LM) samples were collected 24 h postmortem between the 6th thoracic and 5th lumbar vertebrae from the right half of each carcass. The LM between the 6th and 12th thoracic vertebrae was frozen and stored at −20 °C for fatty acid analysis. The remaining portion of LM from the 12th thoracic vertebrae and 5th lumbar vertebrae was packaged in vacuum bags (Combivac, Felzmann, Linz, Austria) using a packaging machine (Minipack–Torre, SpA, MVS–35, Dalmine, Italy) and aged at 4 °C for 72 h. This portion was used for cooking losses and Warner–Bratzler shear force determinations.

### 2.5. Meat Quality Measurements

The pH was assessed at 1 h (pH_1_) and 24 h (pH_24_) postmortem in the LM, between 1st and 2nd lumbar vertebrae, using a pH meter equipped with a penetration electrode and thermometer (Hanna Instruments, HI–9025, Woonsocket, RI, USA). Meat color was measured on the LM surface after 60 min of blooming by placing the samples in containers covered with polyethylene film at 4 °C, using the L* (lightness), a* (redness), and b* (yellowness) color space [18] with a Minolta CR–10 colorimeter (Osaka, Japan). To assess cooking loss, LM samples of about 30 g were placed individually in polyethylene bags in a water bath at 78 °C. The samples were heated until an internal temperature of 75 °C was achieved and monitored with thermocouples. After being cooled for 15 min under running tap water, the samples were stored for 3 h at 4 °C, dried with filter paper, and weighed. Cooking loss was measured by comparing the final with the initial weight and is expressed as a percentage of the initial weight [19]. The meat samples used to determine the cooking loss were then cut into cuboid shape subsamples (3 to 4) of 1 cm^2^ cross-section and 3–4 cm in length to determine the shear force, after room temperature equilibrium, using a Warner–Bratzler rectangular hole probe coupled to a Texture Analyser TA.XT plus texturometer with a load cell of 30 kgF (Stable Micro Systems, Godalming, UK). To perform this analysis, blade velocity and trigger force were set to 120 cm/min and 5 g, respectively, and the subsamples were placed with fibers perpendicular to the direction of the blade. Mean values for maximum shear force (kg/cm^2^) over each subsample group were then obtained.

For fatty acid determination, the procedures described by Argemi-Armengol et al. [20] were followed. Briefly, the LM samples were trimmed of intermuscular and subcutaneous fat before fatty acid (FA) analysis. To determine fat content, the Ankom procedure (AOCS, 2005) [21] (Official Procedure Am 5–04) was applied using an Ankom extractor (XT10; Ankom Technology, Madrid, Spain). The FA methyl esters were obtained by transesterification using a 2% (*v*/*v*) methanol/sulfuric acid solution, with heating for 30 min at 80 °C, centrifugation at 3000 rpm for 5 min, and collection of the final supernatant. The FA methyl esters’ analysis was performed in duplicate via gas chromatography with a 30 m × 0.25 mm capillary column and a flame ionization detector (Agilent DB-23; Agilent Technologies, Santa Clara, CA, USA). The helium was used as the carrier gas at a flow rate of two mL/min. The oven temperature was programmed to increase 35 °C per minute between 150 °C and 180 °C and at 5 °C per minute up to 220 °C. For the injector and detector, it was considered a temperature of 250 °C. The relative percentage of each FA in relation to the total FA was considered. The FAs were identified by comparing the retention times with a known standard Supelco^®^ 37 Component FAME Mix (Supelco, Bellefonte, PA, USA). In total, 34 FAs were detected and quantified. The proportions of saturated fatty acids (SFA) (C10:0; C12:0; C13:0; C14:0; C15:0; C16:0; C17:0; C18:0; C20:0; C21:0; C22:0 and C23:0); polyunsaturated (PUFA) (C18: 2n − 6; C18: 3n − 3; C18: 3n − 6; C20: 2n − 6; C20: 3n − 6; C20: 3n − 3; C20: 4n − 6; C20: 5n − 3; C22: 5n − 3 and C22: 6n − 3); monounsaturated (MUFA) (C14: 1n − 5; C15: 1n − 5; C16: 1n − 7; C17: 1n − 7; C18: 1n − 9; C18: 1n − 7; C20: 1n − 9; C22: 1n − 9); cis/trans (9c,11t-C18:2; 9t,11t-C18:2; t11 C18:1; 9t-C18:1) were calculated.

### 2.6. Statistical Analyses

Data were subjected to an analysis of variance (ANOVA), performed on JMP^®^, version 14 [22]. Diets were used as the main factor. Tukey’s multiple comparison test evaluated significant differences. Significance was declared at *p* < 0.05.

## 3. Results

### 3.1. Live Weight, Carcass Traits, and Meat Quality

In trial 1 and trial 2, no differences (*p* > 0.05) were found for LWS and CCW, although there was a tendency (*p* = 0.06) for dressing (CCW basis) % to be different in trial 1 (Table 2).

For cuts percentage and cuts composition after dissection on both trials (data not shown), no differences were reported. For carcass composition in tissues and muscle:bone ratio, there were also no differences (*p* > 0.05) among diets for either trial 1 or trial 2 (Table 3). 

No effect (*p* > 0.05) of diet on pH, L*a*b* color parameters, and cooking loss was observed. Lambs fed only *Lupinus luteus* (LL20) presented higher shear force values than those fed LL10, hence tougher meat (*p* = 0.045; Table 4). However, the shear force was similar between lamb meat and the rest of the groups. Although pH was not different (*p* > 0.05) between diets in both trials, it is important to point out that pH values were particularly high in the second trial, specially pH1.

### 3.2. Fatty Acids

Table 5 shows fatty acids with values greater than 1% (g/100 g identified FA). For both trials, the fat composition comprises SFA, MUFA, and PUFA fatty acids, with palmitic (C16:0), stearic (C18:0), and oleic (c9 C18:1) fatty acids being the most abundant. Among these FAs, oleic acid (c9 C18:1) showed the highest percentage, ranging from 29% to 36%, followed by palmitic acid (C16:0) (from 20% to 23%) and stearic (C18:0) (from 18% to 23%). In general, diets did not have a significant effect on FAs. However, in trial 1, diets show a significant effect (*p* = 0.002) on t11 C18:1, in which the LL5 diet showed higher values than the LA5 diet and was similar to the control. A similar trend was observed for C16:0. It was also observed that the LM of lambs on the LL5 and LA5 diets showed higher values of oleic MUFAs (*p* < 0.05) than the control (Table 5). 

No differences were found among diets in most FA groups, except for MUFA in trial 1. In this case, diets with lupin inclusion had a higher content (*p* < 0.05) of MUFA fatty acids (Table 6). Regardless, similarly to what was observed for individual FA in all alternative sources of protein diets, the results were very similar to those of the control diet.

## 4. Discussion

Lupin incorporation had no overall effect on carcass characteristics, although dressing percentages of all the carcasses were lower than the ones observed in younger and lighter lambs of the same breed [17,23], as well as on lambs with similar LWS [9,10]. However, it is important to note that lambs in the studies of Santos et al. [23] and Santos et al. [17] were slaughtered around 8–11 kg LW (around weaning) and produced in accordance with the Protected Denomination of 89 Origin (PDO) specifications for carcasses of “Borrego terrincho–PDO”, which might explain the differences in dressing percentages. Dietary lupin incorporation also had no influence (*p* > 0.05) on the percentage of cuts in the carcass, which was reasonably similar to the values observed by other authors [9,10,24]. Overall, muscle content in the carcass was lower than the content previously observed in carcasses of lambs of the same breed, considering the values reported by Santos et al. [17], which, in turn, led to lower muscle:bone ratios than the ratios observed by these authors. This can be explained by the fact that the animals studied by Santos et al. [17] were suckling lambs. Since there were no statistical differences (*p* > 0.05) among groups, this could indicate that the inclusion of lupins in lambs’ diets will not impact the dressing percentages, although this could also be due to the small sample size in this study. Muscle:bone ratios were lower than expected for this breed [17]. Leg muscle:bone ratio, for example, was slightly lower than the 2.64 value reported by Lestingi et al. [10] when incorporating 23% of *Lupinus albus* in Gentile di Puglia lambs’ diets. However, the same study reported lower loin muscle:bone values (1.54), due to a far higher percentage of bone than the one found in this study, which might be attributed to phenotypic differences between the two breeds. 

Particularly in trial 2, muscle pH_24_ was higher than desirable [17] and reported by other authors using lupins in lambs’ diets [25,26]. During the preslaughter period, all lambs were handled by the same person to reduce acute stress as much as possible since it can increase ultimate meat pH [26]. Despite this outcome, there seems to have been no effect of diet on this parameter (*p* > 0.05). Meat color was within the values previously found for this breed [17]. Other authors have reported darker meats than the ones in this study, in crossbred lambs and Merino wether weaner sheep fed diets with 20–35% (DM) lupin incorporation [27,28] (L* = 37.5 ± 0.8; L* = 39.0 ± 0.6, respectively). The values of b* of LL20 lambs (b* = 12.1), which were fed the highest lupin inclusion of both trials, were similar to the ones reported by White et al. [28] with higher lupin inclusions (b* = 12.8). In the present study, lupin inclusion in the diets resulted in slightly tougher lamb meat (4.70 kg/cm^2^; *p* = 0.045), as was previously reported [29] for lambs fed a mixture of barley and lupins. Although Santos et al. [17] reported far lower shear force values in lamb meat from the same breed, the methodology applied by these authors was different and, therefore, not comparable to the values found in this study. Other authors have reported lower shear force values than those in this study [9,24] (2.48 and 2.04 kg/cm^2^, respectively); however, overall, lupin inclusion in lambs’ diets tends not to have an impact on meat tenderness. Higher sample size would probably clarify the results and provide a clearer conclusion than these preliminary results. Since higher meat pH can be associated with higher tenderness [25], this might also explain the present study results. While no differences were found in either trial for cooking losses (*p* > 0.05), the high meat pH values found in the second trial might also justify the lower cooking losses [30] observed in these lambs’ meat (9% vs. 11% in trial 1).

The evaluation of the fatty acid composition of the LM of lambs fed diets containing alternative protein sources muscle has been the target of several researchers [9,31]. Diets including faba beans (*Vicia faba*), peas (*Pisum sativum*), and lupins (*Lupinus* sp.) to replace soybean meal have been tested over the years to understand the effects on the intramuscular FAs of lambs [9,29,32,33]. In the present study, the most abundant fatty acid in the intramuscular fat of lambs was oleic acid (c9 C18:1), which has been positively associated with human health [34,35] and did not differ among treatments except for trial 1, where the incorporation of Lupins proved to have a positive effect on this FA. These results are consistent with those of other studies in which alternative protein sources were used [29,36]. However, reports from other studies testing alternative protein sources show variable effects on this FA. For example, Lestingi et al. [9] found that the meat of lambs fed lupins contained lower levels of oleic acid (c9 C18:1) than peas or the combination of lupin and peas, and Lanza et al. [32] reported that oleic acid in intramuscular fat was higher (*p* < 0.05) in the pea group that that in the soybean-meal group. On the other hand, vaccenic acid (t11 C18:1) showed a higher value (*p* < 0.05) in trial 1 for the group in which *Lupinus albus* was used but had no effect when compared with the groups with *Lupinus luteus* and soybean meal. Differences in vaccenic acid were also verified by other authors who studied alternative legume seeds as a protein alternative to soybean meal [32,37]. The reason for this is unclear, but differences in the fatty acid composition may depend on incomplete ruminal hydrogenation of dietary fat [37].

Unlike the results found in this study, other authors showed differences in PUFAs. For example, Scerra et al. [29] compared diets containing pea, faba bean, and soybean meal and concluded that the meat of lambs fed with peas had higher proportions of the essential fatty acids C18: 2n − 6 and C18: 3n − 3 than those of the other groups. On the other hand, faba beans led to higher proportions of PUFAs (*p* < 0.01) in lamb meat than sweet lupins and peas, according to Gómez-Cortés et al. [33]. Regarding the n − 6/n − 3 fatty acid ratio, the nutritional guidelines for human consumption recommend optimizing the intake of foods containing high amounts of n-3 fatty acids, which is proven to reduce the incidence of cardiovascular diseases [38]. Our results show that the n-6/n-3 fatty acid ratio is similar in meats from lambs fed on lupin and soybean meal diets. Additionally, the values for this ratio are similar to those reported by other authors who tested lupins and other legume grains [9,32]. 

## 5. Conclusions

Lupin incorporation in diets of growing lambs produced similar results to soybean meal in terms of carcass traits and meat physical characteristics. Replacement of soybean meal by lupins in fattening lambs’ diets had minor effects on their intramuscular FA profile. However, adding legume grains such as lupins to lamb diets seemed to have no effect on the n-6/n-3 ratio. Nevertheless, the lack of significant differences could also be attributed to the small sample size. Combined with the results observed during the growth and digestibility trials [14], lupin incorporation in lambs’ diets can present a solution for some producers. Further research should be conducted with a larger sample to provide more information and study the effect of lupin inclusion on the palatability of lambs’ meat. 

## Figures and Tables

**Table 1:** Chemical composition (g/kg DM) and levels of inclusion of diet components (g/kg as fed) of the different treatment diets.

Diets	Diet Components					Chemical Composition		
	Soybean Meal	Wheat	*Lupinus albus* (LA)	*Lupinus luteus* (LL)	Hay	DM	CP	NDF
Trial 1								
C	150	20	0	0	830	930	121	637
LL5	100	20	0	50	830	933	117	629
LA5	100	20	50	0	830	923	114	632
Trial 2								
C	170	20	0	0	810	929	130	626
LL10	100	20	0	100	780	925	127	621
LL15	50	20	0	150	780	937	128	628
LL20	0	20	0	200	780	941	128	628

C—control; LL5—*Lupinus luteus* 50 g/kg; LA5—*Lupinus albus* 50 g/kg; LL10—*Lupinus luteus* 100 g/kg; LL15—*Lupinus luteus* 150 g/kg; LL20—*Lupinus luteus* 200 g/kg; DM—dry matter; CP—crude-protein; NDF—neutral detergent fiber. Table adapted from Almeida et al. [14].

**Table 2:** Least square means (±SE) of live weight at slaughter, cold carcass weight, and carcass dressing of lambs in trials 1 and 2.

Trial	Diets	LWS (kg)	CCW (kg)	Dressing (CCW Basis) %
Trial 1	C (n = 3)	23.8 ± 0.82	9.18 ± 0.35	38.6 ± 1.22
	LA5 (n = 3	24.7 ± 1.56	9.3 ± 0.52	37.7 ± 0.83
	LL5 (n = 3)	23.7 ± 1.46	9.4 ± 0.43	40.0 ± 1.34
*p*		0.471	0.818	0.060
Trial 2	C (n = 3)	21.2 ± 1.96	8.38 ± 0.80	39.5 ± 2.26
	LL10 (n = 4)	21.2 ± 1.75	7.9 ± 0.89	37.0 ± 1.53
	LL15 (n = 3)	22.7 ± 0.54	8.6 ± 0.43	38.1 ± 1.44
	LL20 (n = 4)	22.1 ± 1.38	8.3 ± 0.98	37.5 ± 2.47
*p*		0.483	0.603	0.430

C—control; LL5—*Lupinus luteus* 50 g/kg; LA5—*Lupinus albus* 50 g/kg; LL10—*Lupinus luteus* 100 g/kg; LL15—*Lupinus luteus* 150 g/kg; LL20—*Lupinus luteus* 200 g/kg; LWS—live weight at slaughter; CCW—cold carcass weight.

**Table 3:** Least square means (±SE) of carcass composition in tissues (%) and muscle:bone ratio of lambs in trials 1 and 2.

Trial	Diets	Carcass Tissues (%)				Muscle:Bone
		Muscle	Bone	Intermuscular Fat	Subcutaneous Fat	
Trial 1	C (n = 3)	58.0 ± 1.30	26.1 ± 0.74	11.7 ± 1.37	2.9 ± 0.12	2.0 ± 0.40
	LA5 (n = 3)	58.3 ± 1.92	25.7 ± 1.39	11.0 ± 0.15	3.2 ± 0.76	1.7 ± 0.32
	LL5 (n = 3)	58.2 ± 1.13	25.8 ± 0.77	11.8 ± 0.85	2.7 ± 0.21	2.1 ± 0.36
*p*		0.977	0.870	0.466	0.441	0.366
Trial 2	C (n = 3)	56.0 ± 1.64	31.4 ± 1.42	8.3 ± 2.79	3.8 ± 0.83	1.5 ± 0.15
	LL10 (n = 4)	55.7 ± 2.22	31.4 ± 1.79	7.2 ± 1.99	4.9 ± 0.61	1.3 ± 0.12
	LL15 (n = 4)	57.0 ± 1.22	30.2 ± 1.67	7.9 ± 1.86	4.5 ± 0.60	1.9 ± 0.14
	LL20 (n = 4)	53.6 ± 2.77	31.8 ± 2.72	9.1 ± 2.54	4.8 ± 0.93	1.4 ± 0.28
*p*		0.191	0.690	0.710	0.238	0.339

C—control; LL5—*Lupinus luteus* 50 g/kg; LA5—*Lupinus albus* 50 g/kg; LL10—*Lupinus luteus* 100 g/kg; LL15—*Lupinus luteus* 150 g/kg; LL20—*Lupinus luteus* 200 g/kg.

**Table 4:** Least square means (±SE) of meat quality attributes of lambs in trials 1 and 2.

Trial	Diets	pH		Color			CL (%)	SF (kg/cm^2^)
		pH_1_	pH_24_	L*	a*	b*		
Trial 1	C (n = 3)	6.7 ± 0.06	6.0 ± 0.33	47.1 ± 3.64	16.8 ± 0.93	15.8 ± 0.63	11.6 ± 1.16	4.9 ± 0.40
	LA5 (n = 3)	6.7 ± 0.07	5.8 ± 0.08	46.3 ± 2.13	14.7 ± 1.77	15.0 ± 0.80	11.8 ± 2.38	5.3 ± 0.79
	LL5 (n = 3)	6.7 ± 0.04	5.8 ± 0.10	46.0 ± 2.71	15.5 ± 0.57	15.4 ± 1.06	11.2 ± 2.03	5.2 ± 0.54
*p*		0.759	0.147	0.876	0.157	0.388	0.934	0.739
Trial 2	C (n = 3)	6.8 ± 0.63	6.6 ± 0.36	45.3 ± 2.73	16.1 ± 0.24	13.7 ± 0.77	8.5 ± 0.94	3.9 ± 1.18 ^ab^
	LL10 (n = 4)	7.4 ± 0.27	6.4 ± 0.15	46.4 ± 4.10	14.3 ± 2.15	13.9 ± 1.10	9.1 ± 1.48	3.8 ± 0.89 ^b^
	LL15 (n = 4)	7.8 ± 0.49	6.4 ± 0.11	45.8 ± 1.60	16.3 ± 2.20	14.7 ± 0.78	9.4 ± 2.59	4.2 ± 0.92 ^ab^
	LL20 (n = 4)	7.3 ± 0.42	6.5 ± 0.43	44.9 ± 3.35	15.1 ± 1.98	12.8 ± 1.32	8.7 ± 1.49	4.7 ± 1.09 ^a^
*p*		0.477	0.860	0.915	0.475	0.144	0.728	0.045

C—control; LL5—*Lupinus luteus* 50 g/kg; LA5—*Lupinus albus* 50 g/kg; LL10—*Lupinus luteus* 100 g/kg; LL15—*Lupinus luteus* 150 g/kg; LL20—*Lupinus luteus* 200 g/kg; CL—cooking losses; SF—shear force. Different superscript letters (a, b) on the same column indicate significant differences (*p* < 0.05).

**Table 5:** Fatty acid (FA) composition (g/100 g identified FA) in LM of lambs affected by diets in trials 1 and 2.

Trial	Diets	SFA				MUFA					PUFA	
		C14:0	C16:0	C17:0	C18:0	C15:1n − 5	C16:1n − 7	C18:1n − 7	t11 C18:1	c9 C18:1	C18:2n − 6	C20:4n − 6
Trial 1	C (n = 3)	3.81	22.7 ^a^	1.56	17.86	1.57	1.25	1.14	1.78 ^a^	32.3 ^b^	6.42	2.76
	LA5 (n = 3)	4.02	21.6 ^b^	1.38	17.17	1.31	1.23	1.02	1.40 ^b^	35.7 ^a^	6.28	2.42
	LL5 (n = 3)	3.19	22.4 ^a^	1.42	18.24	1.36	1.29	0.98	1.86 ^a^	34.2 ^a^	5.95	2.74
*p*		0.577	0.019	0.554	0.837	0.588	0.960	0.772	0.002	0.013	0.839	0.707
Trial 2	C (n = 3)	3.29	19.7	1.40	18.1	2.70	0.98	1.26	1.73	29.1	9.27	5.38
	LL10 (n = 4)	2.24	20.4	1.52	23.1	1.67	0.89	0.92	1.48	30.8	7.01	3.70
	LL15 (n = 4)	2.93	20.3	1.33	20.0	1.96	1.02	0.97	1.64	31.3	8.14	3.97
	LL20 (n = 4)	3.17	19.5	1.21	20.6	2.06	1.08	0.93	1.55	30.0	7.96	4.97
*p*		0.512	0.898	0.208	0.268	0.222	0.824	0.064	0.806	0.750	0.126	0.496

C—control; LA5—*Lupinus albus* 50 g/kg; LL5—*Lupinus luteus* 50 g/kg; LL10—*Lupinus luteus* 100 g/kg; LL15—*Lupinus luteus* 150 g/kg; LL20—*Lupinus luteus* 200 g/kg; FA—fatty acid; SFA—saturated FA; MUFA—monounsaturated FA; PUFA—polyunsaturated FA. Different superscript letters (a, b) on the same column indicate significant differences (*p* < 0.05).

**Table 6:** Groups of SFA, MUFA and PUFA (g/100 g identified FA) in LM of lambs affected by diets in trials 1 and 2.

Trial	Diets	SFA	MUFA	PUFA	n − 3	n − 6	n − 6/n − 3
Trial 1	C (n = 3)	47.6	39.4 ^b^	12.8	2.18	10.1	4.73
	LA5 (n = 3)	45.8	42.2 ^a^	12.1	2.04	9.45	4.64
	LL5 (n = 3)	46.7	41.2 ^a^	12.1	2.01	9.48	4.72
*p*		0.492	0.031	0.776	0.541	0.865	0.985
Trial 2	C (n = 3)	44.0	37.2	18.6	2.34	15.75	6.74
	LL10 (n = 4)	48.8	37.1	14.1	1.95	11.69	6.08
	LL15 (n = 4)	46.1	38.2	15.5	1.99	13.04	6.67
	LL20 (n = 4)	46.2	37.1	16.6	2.00	14.05	7.08
*p*		0.200	0.943	0.353	0.781	0.357	0.093

C—control; LA5—*Lupinus albus* 50 g/kg; LL5—*Lupinus luteus* 50 g/kg; LL10—*Lupinus luteus* 100 g/kg; LL15—*Lupinus luteus* 150 g/kg; LL20—*Lupinus luteus* 200 g/kg; FA—fatty acids; SFA—saturated FA; MUFA—monounsaturated FA; PUFA—polyunsaturated FA; LC—long chain. Different superscript letters (a, b) on the same column indicate significant differences (*p* < 0.05).

## Data Availability

The data presented in this study is available on request.
